# *Helicobacter pylori* isolated from Iranian drinking water: *vacA*,* cagA*,* iceA*,* oipA* and *babA2* genotype status and antimicrobial resistance properties

**DOI:** 10.1002/2211-5463.12054

**Published:** 2016-04-04

**Authors:** Reza Ranjbar, Faham Khamesipour, Nematollah Jonaidi‐Jafari, Ebrahim Rahimi

**Affiliations:** ^1^ Molecular Biology Research CenterBaqiyatallah University of Medical SciencesTehranIran; ^2^ Cellular and Molecular Research CenterSabzevar University of Medical SciencesSabzevarIran; ^3^ Health Research CenterBaqiyatallah University of Medical SciencesTehranIran; ^4^ Department of Food HygieneFaculty of Veterinary MedicineShahrekord BranchIslamic Azad UniversityShahrekordIran

**Keywords:** antibiotic resistance properties, drinking water, genotypes, genotyping, *Helicobacter pylori*, Iran

## Abstract

Despite the clinical importance of *Helicobacter pylori* in human gastric disorders, its exact route of transmission is still uncertain. Based on the contentious hypothesis and findings of previous investigations, water may play an important role in the transmission of *H. pylori* to humans. This study was carried out to investigate the *vacA*,* cagA*,* oipA*,* iceA* and *babA2* genotype status and antimicrobial resistance properties of *H. pylori* strains isolated from the drinking water samples of four major provinces in Iran. A total of 400 drinking water samples were cultured and tested. *H. pylori*‐positive strains were analyzed for the presence of various genotypes and antimicrobial resistance. Twelve of 400 (3%) water samples were positive for *H. pylori*. Samples from Isfahan province had the highest, while those from Shiraz had the lowest prevalence of *H. pylori*. The seasonal distribution was also determined, with the highest prevalence of bacteria in the summer season (7.36%). *H. pylori* strains harbored the highest levels of resistance against ampicillin (100%), erythromycin (75%), clarithromycin (75%), and trimethoprim (58.3%). The most commonly detected genotypes were *vacAs1a* (83.3%), *vacAm1a* (66.6%), *vacAs2* (50%) and *cagA* (50%). The presence of similar genotypes in the *H. pylori* strains of drinking water and those of human clinical samples suggest that contaminated water maybe the sources of bacteria. Spiramycin and furazolidone are suggested for the treatment of cases of *H. pylori* infection.

**Abbreviations**
AM: ampicillin
AMX: amoxicillin
*babA*: blood group antigen‐binding adhesion
*cag*: cytotoxin‐associated gene
Cef: cefsulodin
CLR: clarithromycin
CLSI: Clinical Laboratory Standards Institute
ER: erythromycin
FZL: furazolidone
*H. pylori*: *Helicobacter pylori*
*iceA*: induced by contact with the epithelium antigen
Lev: levofloxacin
Met: metronidazole
*oip*: outer inflammatory protein
RIF: rifampin
Spi: spiramycin
TRP: trimethoprim
*vacA*: vacuolating cytotoxin

*Helicobacter pylori* is a gram‐negative, microaerophilic and spiral‐shaped bacterium that efficiently colonizes the human gastric mucosa 1. Bacterial colonization of the gastric mucosa is the main cause of ulcers in the stomach and duodenum 1. *H. pylori* is also known as a causative agent of peptic ulcer disease, type B gastritis, gastric adenocarcinoma, and mucosa‐associated lymphoid tissue lymphoma 1, 2. It has been estimated that 17–86% of hospitalized patients with peptic ulcers were infected with *H. pylori* 2, 3, 4. Documented data showed that the *H. pylori* colonizes more than 50% of the world's population 5. Despite the high incidence of bacteria in human populations, no reservoir outside of the human stomach has been identified 6. Transmission presumably occurs through fecal–oral and oral–oral routes. One of the most commonly reported reservoir of the *H. pylori* outside of human stomach is contaminated water 7, 8. An epidemiological association between water sources and the prevalence of *H. pylori* infection has been identified 8. The hypothesis of water being a route of transmission of *H. pylori* 8, 9, 10 is supported by epidemiologic studies that have observed a higher prevalence of *H. pylori* infection in developing countries which suffered from problems related to the sanitary distribution of water among the population 11.

To appraise the pathogenicity of *H. pylori* especially in possible sources of transmission like water, evaluation of latent virulence factors and genotypes is essential. The most commonly important virulence factors among *H. pylori* strains of different clinical outcomes of human and animal beings are the vacuolating cytotoxin (*vacA*), induced by contact with the epithelium antigen (*iceA*), cytotoxin‐associated gene (*cag*), blood group antigen‐binding adhesion (*babA*), and outer inflammatory protein (*oip*) 12, 13, 14, 15, 16. These genes are usually induced by adhesion and invasion to the gastric epithelial cells 12, 13, 14, 15, 16. Genotyping using these virulence markers is considered as one of the best approaches to determinate correlations between *H. pylori* isolates from different samples.

In order to appraise the pathogenicity of *H. pylori*, study the antimicrobial resistance properties is another important point. Treatment is a critical point in the epidemiology of *H. pylori* infection in humans, since therapeutic options have become somewhat limited because of the presence of multidrug‐resistant strains of this bacterium 14, 15, 16, 17. Moreover, to the best of our knowledge, we could not find any published data on the antibiotic resistance pattern of *H. pylori* strains isolated from drinking water samples.

Data on the epidemiology and transmission of *H. pylori* is extremely significant in order to prevent its distribution and to identify high‐risk populations, especially in areas that have high rates of gastritis, peptic ulcers, and gastric cancer such as Iran 13, 17, 18, 19. Considering the unclear epidemiological aspects of *H. pylori* in Iranian drinking water sources, the present investigation was carried out in order to study the exact status of *vacA*,* cagA*,* iceA*,* oipA* and *babA2* genotypes and the antibiotic resistance patterns of *H. pylori* isolates from drinking water samples.

## Materials and methods

### Sample collection

From January 2014 to January 2015, overall 400 drinking water samples were collected from the various parts of Isfahan, Shiraz, Yazd, and Shahrekord province, Iran. All samples were collected from various seasons of the year including summer (*n* = 95), autumn (*n* = 100), winter (*n* = 110), and spring (*n* = 95). Samples (100 mL in 1000‐mL glass bottles containing 0.5 g of sodium thiosulphate for dechlorination) were transported to the lab on ice, and used within 2 h of collection. All samples were collected in aseptic conditions away from any cross‐contamination in separate glass bottles. Drinking water samples of these major cities of Iran were treated using chlorination.

### Isolation of *H. pylori*

Samples were filtered through 0.45‐μm filter membrane (Albet Co., Barcelona, Spain). Each membrane was then immersed into 2 mL of Tryptic Soy Broth (TSB, Merck, Darmstadt, Germany) for 1 h. After that, each 2 mL TSB was taken and cultured for *H. pylori*. Samples were cultured on Brucella agar (Merck) containing campylobacter‐selective supplement (5 mg·L^−1^, Merck), trimethoprim (0.25 mg·L^−1^), colistin methanesulfonate (30 mg·L^−1^), cycloheximide (100 mg·L^−1^), nalidixic acid (30 mg·L^−1^), trimethoprim (30 mg·L^−1^), vancomycin (10 mg·L^−1^) (Sigma, St Louis, MO, USA), amphotericin B (10 mg·L^−1^), sheep blood (5%), and 7% fetal calf serum (Sigma). After 72 h incubation at 37 °C in microaerophilic condition (85% N_2_, 10% CO_2_ and 5% O_2_,) using MART system (Anoxamat, Lichtenvoorde, The Netherlands), the bacterial growth was tested and confirmed as *H. pylori* using Gram staining, urease, and oxidase tests. For comparison, a reference strain of *H. pylori* (ATCC 43504) was employed.

### Antimicrobial susceptibility testing

Pure cultures of *H. pylori* were applied for antibiotic susceptibility test. One strain from each *H. pylori*‐positive sample was selected for this aim. Antimicrobial susceptibility test was accomplished by the Kirby–Bauer disk diffusion method using Mueller–Hinton agar (Merck) supplemented with 5% defibrinated sheep blood and 7% fetal calf serum, according to the Clinical Laboratory Standards Institute (CLSI, 2012) 20. The antimicrobial resistance of *H. pylori* was measured against the widely used antibiotics in cases of *H. pylori* gastric ulcer. The following antimicrobial disks (HiMedia Laboratories, Mumbai, India) were used: ampicillin (10 μg), metronidazole (5 μg), erythromycin (5 μg), clarithromycin (2 μg), amoxicillin (10 μg), levofloxacin (5 μg), rifampin (30 μg), cefsulodin (30 μg), trimethoprim (25 μg), furazolidone (1 μg) and spiramycin (100 μg). After incubation at 37 °C for 48 h in a microaerophilic atmosphere (85% N_2_, 10% CO_2_, and 5% O_2_,), the susceptibility of the *H. pylori* was measured against each antimicrobial agents. Results were construed in accordance with interpretive criteria provided by CLSI 20. The *H. pylori* ATCC 43504 was used as quality control organisms in antimicrobial susceptibility determination.

### DNA extraction and *H. pylori* 16S rRNA gene amplification

Suspected colonies were also identified as *H. pylori* based on the PCR technique. Genomic DNA was extracted from the colonies with typical characters of *H. pylori* using a DNA extraction kit for cells and tissues (Roche Applied Science, Mannheim, Germany, 11814770001) according to the manufacturer's instructions and its concentration was assessed by optic densitometry. Extracted DNA was amplified for the *16S rRNA* gene (primers: HP‐F: 5′‐CTGGAGAGACTAAGCCCTCC‐3′ and HP‐R: 5′‐ATTACTGACGCTGATTGTGC‐3′) 21. PCR reactions were performed in a final volume of 50 μL containing 5 μL 10× buffer (Fermentas, Mannheim, Germany) + MgCl_2_, 2 mm dNTP (Fermentas), 2 units of Taq DNA polymerase (Fermentas), 100 ng genomic DNA as a template, and 25 picomole of each primer. PCR was performed using a thermal cycler (Eppendorf Co., Hamburg, Germany) under the following conditions: an initial denaturation for 2 min at 94 °C; 30 cycles of 95 °C for 30 s, 60 °C for 30 s, and 72 °C for 30 s and a final extension at 72 °C for 8 min. *H. pylori* (ATCC 43504) was employed as a positive control in this part of the study.

### Genotyping of vacA, cagA, iceA, babA2, and oipA genotypes in the *H. pylori* isolates of drinking water

The genotype data refer to pooled colonies (individual water samples) and not individual isolates of *H. pylori*. The presence of the *iceA1*,* iceA2*,* oipA*,* cagA*,* babA2* genotypes and also various genotypes of *vacA* alleles (*s1a*,* s1b*,* s1c*,* m1a*,* m1b* and *m2*) were determined using PCR technique. List of primers and PCR program are shown in Table 1 22, 23, 24, 25, 26, 27, 28, 29, 30. PCR amplifications were performed in a programmable thermal cycler (Master Cycle Gradiant; Eppendorf) and all runs included one negative DNA control consisting of PCR grade water and two or more positive controls (26695, J99, SS1, Tx30, 88‐23 and 84–183).

**Table 1:** Oligonucleotide primers and PCR conditions used for genotyping of *Helicobacter pylori* strains isolated from Iranian drinking water samples 22, 23, 24, 25, 26, 27, 28, 29, 30

Genes	Primer sequence (5′–3′)	Size of product (bp)	Volume of PCR reaction (50 μL)	PCR programs
*vacA s*_*1*_ *a*	F: CTCTCGCTTTAGTAGGAGC R: CTGCTTGAATGCGCCAAAC	213		
*vacA s*_*1*_ *b*	F: AGCGCCATACCGCAAGAG CTGCTTGAATGCGCCAAAC	187		
*vacA s*_*1*_ *c*	F: CTCTCGCTTTAGTGGGGYT R: CTGCTTGAATGCGCCAAAC	213	5 μL PCR buffer 10× (Fermentas) 1.5 mm MgCl_2_ 200 μm dNTP (Fermentas) 0.5 μm of each primers F and R 1.25 U Taq DNA polymerase (Fermentas) 2.5 μL DNA template	1 cycle: 95 °C for 1 min 32 cycle: 95 °C for 45 s 64 °C for 50 s 72 °C for 70 s 1 cycle: 72 °C for 5 min
*vacA s*_*2*_	F: GCTAACACGCCAAATGATCC R: CTGCTTGAATGCGCCAAAC	199		
*vacA m*_*1*_ *A*	F: GGTCAAAATGCGGTCATGG R: CCATTGGTACCTGTAGAAAC	290		
*vacA m*_*1*_ *B*	F: GGCCCCAATGCAGTCATGGA R: GCTGTTAGTGCCTAAAGAAGCAT	291		
*vacA m*_*2*_	F: GGAGCCCCAGGAAACATTG R: CATAACTAGCGCCTTGCA	352		
*cag A*	F: GATAACAGCCAAGCTTTTGAGG R: CTGCAAAAGATTGTTTGGCAGA	300	5 μL PCR buffer 10× (Fermentas) 2 mm MgCl_2_ 150 μm dNTP (Fermentas) 0.75 μm of each primers F and R 1.5 U Taq DNA polymerase (Fermentas) 3 μL DNA template	1 cycle: 94 °C for 1 min 32 cycle: 95 °C for 60 s 56 °C for 60 s 72 °C for 60 s 1 cycle: 72 °C for 10 min
*iceA*_*1*_	F: GTGTTTTTAACCAAAGTATC R: CTATAGCCASTYTCTTTGCA	247	5 μL PCR buffer 10× (Fermentas) 2 mm MgCl_2_ 200 μm dNTP (Fermentas) 0.5 μm of each primers F and R 1.5 U Taq DNA polymerase (Fermentas) 5 μL DNA template	1 cycle: 94 °C for 1 min 32 cycle: 94 °C for 60 s 56 °C for 60 s 72 °C for 60 s 1 cycle: 72 °C for 8 min
*iceA*_*2*_	F: GTTGGGTATATCACAATTTAT R: TTRCCCTATTTTCTAGTAGGT	229/334		
*oip A*	F: GTTTTTGATGCATGGGATTT R: GTGCATCTCTTATGGCTTT	401	5 μL PCR buffer 10× (Fermentas) 2.5 mm MgCl_2_ 200 μm dNTP (Fermentas) 0.5 μm of each primers F and R 2 U Taq DNA polymerase (Fermentas) 3 μL DNA template	1 cycle: 94 °C for 2 min 32 cycle: 94 °C for 60 s 56 °C for 60 s 72 °C for 60 s 1 cycle: 72 °C for 10 min
*BabA2*	F: CCAAACGAAACAAAAAGCGT R: GCTTGTGTAAAAGCCGTCGT	271	5 μL PCR buffer 10× (Fermentas) 1.5 mm MgCl_2_ 200 μm dNTP (Fermentas) 0.5 μm of each primers F and R 1.25 U Taq DNA polymerase (Fermentas) 2.5 μL DNA template	1 cycle: 95 °C for 1 min 30 cycle: 91 °C for 60 s 45 °C for 60 s 72 °C for 60 s 1 cycle: 72 °C for 8 min

### Gel electrophoresis

The PCR amplification products (10 μL) were subjected to electrophoresis in a 2% agarose gel in 1× TBE buffer (Fermentas) at 80 V for 30 min, stained with ethidium bromide, and images were obtained in a UVIdoc gel documentation systems (UK). The PCR products were identified by 100 bp DNA size marker (Fermentas).

### Statistical analysis

Data were transferred to Microsoft Excel spreadsheet (Microsoft Corp., Redmond, WA, USA) for analysis. Using spss 16.0 statistical software (SPSS Inc., Chicago, IL, USA), Chi‐square test and Fisher's exact two‐tailed test were performed and differences were considered significant at values of *P* < 0.05. The distribution of *H. pylori* genotypes isolated from drinking water was statistically analyzed.

## Results

**Figure 1:**
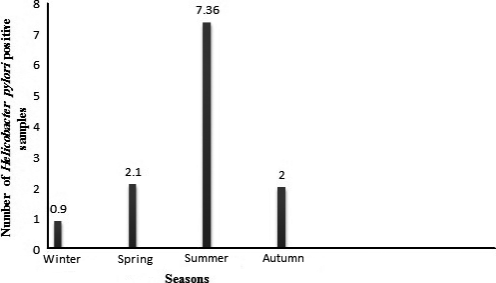
Seasonal distribution of *Helicobacter pylori* in Iranian drinking water samples. The number of samples collected in summer, autumn, winter, and spring seasons were 95, 100, 110 and 95, respectively. Number of positive strains obtained from the water samples collected from summer, autumn, winter, and spring seasons were seven, two, one, and two strains, respectively. Prevalence of *H. pylori* in each season is shown by percentage.

A total of 400 drinking water samples were studied for the presence of *H. pylori*, its genotypes and antimicrobial resistance properties. Table 2 shows the total distribution of *H. pylori* in the drinking water samples of four major province of Iran. Of 400 drinking water samples collected, 12 samples (3%) were contaminated with *H. pylori*. The results of culture method were confirmed using the *16S rRNA*‐based PCR technique. The water samples of Isfahan province were the most contaminated, while those of Shiraz province were less contaminated. Statistically significant difference was seen between the distributions of *H. pylori* and zone of sample collection (*P* < 0.05). Seasonal distribution of *H. pylori* in the drinking water samples of various parts of Iran is shown in Fig. 1. We found that the drinking water samples of summer seasons had the highest levels of *H. pylori*‐contamination (7.36%), followed by spring and autumn (2.1% and 2% respectively). Statistically significant difference was seen between the distributions of *H. pylori* and season of sample collection (*P* < 0.05).

**Table 2:** Total distribution of *Helicobacter pylori* in the drinking water samples in four major provinces of Iran

Province	No. of samples collected	No. of *H. pylori*‐positive samples (%)	No. of *H. pylori*‐positive samples confirmed by PCR (%)
Isfahan	120	5 (4.16)	5 (4.16)
Shiraz	110	2 (1.8)	2 (1.8)
Yazd	100	3 (3)	3 (3)
Shahrekord	70	2 (2.8)	2 (2.8)
Total	400	12 (3)	12 (3)

The results of antimicrobial resistance patterns of *H. pylori* isolates of Iranian drinking water samples is shown in Table 3. *H. pylori* strains of our investigation harbored the highest levels of resistance against ampicillin (100%), erythromycin (75%), clarithromycin (75%) and trimethoprim (58.3%) antimicrobial agents. There were statistically significant differences in the levels of antibiotic resistance between ampicillin and rifampin (*P* = 0.022), ampicillin and cefsulodin (*P* = 0.028), clarithromycin and furazolidone (*P* = 0.033), ampicillin and spiramycin (*P* = 0.034), and amoxicillin and furazolidone (*P* = 0.029).

**Table 3:** Antimicrobial resistance pattern of *Helicobacter pylori* isolates from Iranian drinking water samples

Types of Samples (no. positive results)	Pattern of antibiotic resistance (%)										
	AM10^a^	Met5	ER5	CLR2	AMX10	Lev5	RIF30	Cef30	TRP25	FZL1	Spi100
Isfahan (5)	5 (100)	3 (60)	4 (80)	4 (80)	3 (60)	3 (60)	2 (40)	2 (40)	3 (60)	1 (20)	1 (20)
Shiraz (2)	2 (100)	1 (50)	2 (100)	2 (100)	1 (50)	1 (50)	1 (50)	1 (50)	1 (50)	–	–
Yazd (3)	3 (100)	1 (33.3)	2 (66.6)	2 (66.6)	1 (33.3)	1 (33.3)	–	–	2 (66.6)	–	–
Shahrekord (2)	2 (100)	1 (50)	1 (50)	1 (50)	1 (50)	1 (50)	–	–	1 (50)	–	1 (50)
Total (12)	12 (100)	6 (50)	9 (75)	9 (75)	6 (50)	6 (50)	3 (25)	3 (25)	7 (58.3)	1 (8.3)	2 (16.6)

a AM10: ampicillin (10 μg), Met5: metronidazole (5 μg), ER5: erythromycin (5 μg), CLR2: clarithromycin (2 μg), AMX10: amoxicillin (10 μg), Lev5: levofloxacin (5 μg), RIF30: rifampin (30 μg), Cef30: cefsulodin (30 μg), TRP25: trimethoprim (25 μg), FZL1: furazolidone (1 μg), and Spi100: spiramycin (100 μg).

Distribution of various genotypes of *vacA* alleles, *cagA*,* iceA1*,* iceA2*,* oipA* and *babA2* is shown in table 4. The most commonly detected genotypes among the *H. pylori* isolates of drinking water were *vacAs1a* (83.3%), *vacAm1a* (66.6%), *vacAs2* (50%) and *cagA* (50%). The prevalence of *iceA1*,* iceA2*,* oipA* and *babA2* genotypes were 41.6%, 16.6%, 33.3% and 16.6%, respectively.

**Table 4:** Total distribution of various genotypes in *Helicobacter pylori* strains of Iranian drinking water samples

Types of samples (no. of positive results)	Distribution of various genotypes (%)											
	*s1a*	*s1b*	*s1c*	*s2*	*m1a*	*m1b*	*m2*	*cagA*	*iceA1*	*iceA2*	*oipA*	*babA2*
Isfahan (5)	4 (80)	2 (40)	1 (20)	3 (60)	4 (80)	2 (40)	2 (40)	3 (60)	2 (40)	1 (20)	2 (40)	1 (20)
Shiraz (2)	2 (100)	1 (50)	–	1 (50)	1 (50)	1 (50)	1 (50)	1 (50)	1 (50)	–	–	–
Yazd (3)	2 (66.6)	1 (33.3)	–	1 (33.3)	2 (66.6)	–	1 (33.3)	1 (33.3)	1 (33.3)	1 (33.3)	1 (33.3)	1 (33.3)
Shahrekord (2)	2 (100)	1 (50)	–	1 (50)	1 (50)	1 (50)	1 (50)	1 (50)	1 (50)	–	1 (50)	–
Total (12)	10 (83.3)	5 (41.6)	1 (8.3)	6 (50)	8 (66.6)	4 (33.3)	5 (41.6)	6 (50)	5 (41.6)	2 (16.6)	4 (33.3)	2 (16.6)

## Discussion

The present investigation was carried out to study the prevalence of *H. pylori* in the drinking water samples of four major provinces in Iran as well as to determine the *vacA*,* cagA*,* iceA*,* oipA* and *babA2* genotype status and antibiotic resistance properties of bacterial isolates. Our results showed that 3% of drinking water samples were contaminated with *H. pylori*. Although the prevalence of bacterium is low, according to daily and high consumption of water, it is very impressive. Despite the results of a previous study which revealed that viable helicobacters were not detected in any of the 151 samples from the United Kingdom 31, the results of our investigation showed that all of the *H. pylori* isolates of drinking water were viable in culture media. Bahrami *et al*. 32 tried to detect *H. pylori* in city water, dental units’ water, and bottled mineral water of Iran. Their results showed that the prevalence of *H. pylori* in 2 of 50 tap water samples (4%), 2 of 35 dental units’ water (5.8%) samples, and 1 of 40 water coolers in public places (3%) were contaminated with *H. pylori*. Some possible reasons for the high prevalence of *H. pylori* in drinking water samples in Iran are (a) the lack of efficient methods for water purification, (b) using river water for drinking in some areas of Iran like Isfahan and Yazd province, (c) the possibility of the presence of bacterial colonies as a biofilm in the pipes used for water transfer, (d) application of open water accumulation sources in some areas like Shahrekord and Shiraz, (e) the possibility of leakage of household, industrial, and agricultural wastewater to the sources of drinking water, and finally (f) lack of personal hygiene of refinery rooms’ staff. One possible explanation for the higher prevalence of *H. pylori* in the drinking water of Isfahan province is due to primary contamination of the Zayande‐rood River. The Zayande‐rood River is the main source of drinking water supply of the Isfahan province. This river comes from the Zagros Mountains. After passing through several towns, agricultural lands, and industrial areas, the river reaches the Isfahan steel company and then enters the Isfahan purification facility. Several sources of pollution including Isfahan steel company, towns, villages, industrial factories, and agriculture exist along the path of this river. Entrance of industrial, agricultural, urban and rural waste, and waste waters into the Zayande‐rood River are the main source of water contamination. In addition to Isfahan, the Zayande‐rood River are the main source of water supply for Yazd province. Therefore, primary contamination of Zayande‐rood River and weak performance of refinery rooms are two important factors which support the significant presence of *H. pylori* in the drinking water of Yazd province. Despite our finding, no *H. pylori* was found in the water sources of several studies 11, 33, 34. This could be attributed to the fact that *H. pylori* can survive for a short period of time in water 33. Moreover, the method employed for *H. pylori* isolation may lack sufficient sensitivity to recover very low numbers of *H. pylori* 33, 35, 36.

Marked seasonality with the high prevalence in summer season was seen in the *H. pylori* strains of drinking water of our study. The main reason for the highest prevalence of *H. pylori* strains in summer in Iran is the fact that during this time climatic events, heat, rain, and thunderstorms, as well as variation in barometric pressure may have influence on the prevalence of bacteria. After analyzing the average temperatures of these four seasons in Isfahan province (17 °C for spring, 32 °C for summer, 16 °C for autumn and 5 °C for winter), it was determined that the prevalence rate of *H. pylori* strains in each season is related with their average temperatures. Yahaghi *et al*. 15 reported the similar seasonal distribution of the *H. pylori* strains in vegetable and salad samples. They showed statistically significant differences in the incidence of *H. pylori* between hot and cold seasons of the year.

The results of our study revealed that the presence of *H. pylori* in drinking water could be associated with clinical infections. It is because of the high presence of resistant and virulent strains of *H. pylori* in Iranian drinking water samples. We found that the bacterial strains of our investigation harbored the high levels of resistance against ampicillin (100%), erythromycin (75%), clarithromycin (75%), trimethoprim (58.3%), metronidazole (50%), amoxicillin (50%) and levofloxacin (50%) antimicrobial agents. Similar findings have been reported previously by Thyagarajan *et al*. 37, Yahaghi *et al*. 15, Bang *et al*. 38, and Secka *et al*. 39. Bang *et al*. 38 reported that the *H. pylori* isolates of human clinical samples were highly resistant to metronidazole (34.7%), clarithromycin (16.7%), and amoxicillin (11.8%). Mirzaei *et al*. 40 reported that the prevalence of resistance of *H. pylori* isolates of Iranian clinical samples against metronidazole, clarithromycin, and amoxicillin were 56.3%, 14.6% and 4.2%, respectively. Previous study which was conducted on drinking water showed that the *H. pylori* isolates were resistant against metronidazole (36.4%), clarithromycin (0.9%), amoxicillin (0%), tetracycline (1.8%) and furazolidone (4.5%) 41. Epidemiological investigations of Iran, Nigeria, India, Senegal, China, Saudi Arabia, Taiwan, Colombia, Thailand, Brazil, Egypt and Argentina showed that the *H. pylori* isolates of human clinical specimens had the highest levels of resistance against metronidazole, amoxicillin, quinolones, and tetracycline (WGO 42) which was similar to our results.

The prevalence of resistance against human‐based antimicrobial agents in the *H. pylori* strains of drinking water samples could indirectly confirm the human‐based routes of water infections. One possible explanation for the high prevalence of resistance against ampicillin (100%), erythromycin (75%), clarithromycin (75%), trimethoprim (58.3%), metronidazole (50%), amoxicillin (50%) and levofloxacin (50%) antimicrobial agents in our study maybe the excessive and indiscriminate prescription of these antibiotics in the treatment of cases of *H. pylori* infections in Iran. The possibility of considering spiramycin and furazolidone antibiotics as an alternative for treatment of *H. pylori* could be suggested in Iranian cases of infections. We found impressive high percentage of resistances to clarithromycin (75%), ampicillin (100%), and amoxycillin (50%), which were much higher than those reported by other investigations worldwide 40, 41, 42. These antibiotics are one of the first‐choice treatment agents for *H. pylori* infection and the high prevalence of resistance against these antibiotics are due to the irregular, intense, and illegal prescription of clarithromycin not only for the cases of *H. pylori* infections but also for all types of infectious diseases of the digestive tract. This matter has serious country‐based concern.

Genotyping of *H. pylori* isolates showed that in the water samples of all studied areas of Iran, *vacAs1a* (83.3%), *vacAm1a* (66.6%), *vacAs2* (50%) and *cagA* (50%) genotypes were high. As far as we know, only one study tried to study the genotype status of *H. pylori* strains of untreated municipal waste water samples 43. The results of this study showed the high presence of *vacAs1a*,* vacAm1a*, and vacAs1am1a. High presence of *vacAs1a*,* vacAm1a*,* vacAs2*,* cagA* alleles and also *s1am1a*,* s2m1a*,* s1bm1a*,* s1bm1b*,* s1a m2*,* s2m2* and *m1am2* genotypes in the *H. pylori* strains of human clinical samples such as gastric biopsy, feces, and saliva have been reported previously 44, 45, 46.

Total prevalence of *iceA1*,* iceA2*,* oipA* and *babA2* genotypes in the *H. pylori* strains of our survey were 41.6%, 16.6%, 33.3% and 16.6%, respectively. In a study which was conducted in Iran on human clinical samples 47, the prevalence of *cagA*,* iceA1*,* iceA2*,* oipA* and *babA2* genotypes were 62.2%, 48.6%, 16.2%, 81.1% and 94.6%, respectively. Similarity in the genotyping pattern of *H. pylori* in all provinces of our study and its close proximity with those of human clinical samples of other investigations have indirectly shown the human‐based contamination of drinking water samples in these areas of Iran.

*Helicobacter pylori* strains carrying the s1m1 mosaic combination of the gene *vacA* exhibit higher levels of cytotoxic activity than s1m2 strains, while s2m2 strains secrete the toxin with low or no vacuolating activity *in vitro* and is rarely associated with gastric disease 13, 14, 15, 48. The severity of diseases caused by strains which express *babA* is greater than diseases by strains that do not express the gene 49. The expression of *iceA1* is upregulated on contact between *H. pylori* and human epithelial cells, and may be related with peptic ulcer disease. The expression of *oipA* is associated with IL‐8 induction and is related with severe clinical outcomes 13, 14, 15, 48.

With respect to the high levels of similarities in the *H. pylori* strains of drinking water of our study and those of other investigations which were conducted on clinical samples, it could be concluded that contaminated water may be the sources of *H. pylori* infection for humans. However, there are significant differences between the drinking water isolates and those from patients: resistance to clarithromycin and amoxicillin was much higher in the drinking water isolates than those found in Iranian clinical samples, and the presence of *oipA‐* and *babA2*‐positive strains was much higher in clinical isolates than those from the drinking water in this study. The main reasons for the above findings are higher resistance of *H. pylori* strains of water samples than those of clinical isolates.

## Conclusion

In conclusion, drinking water samples in Iran harbor *H. pylori* similar in genotypes of *vacA*,* cagA*,* iceA*,* oipA* and *babA2* alleles with isolates recovered from various types of human clinical samples. High prevalence of *H. pylori* in our samples suggest that contaminated drinking water in these area of Iran maybe the sources of the bacteria and that it entered the human population after a period of time. There was no high diversity in the genotyping pattern of *H. pylori* between the different areas of Iran which may have shown that there was one source of contamination for drinking water. Prescription of spiramycin and furazolidone antibiotics as an alternative approach for treatment of *H. pylori* could be suggested.

## Author contributions

All authors contributed equally to this work. All authors read and approved the final manuscript.
